# Polymerized human hemoglobin facilitated modulation of tumor oxygenation is dependent on tumor oxygenation status and oxygen affinity of the hemoglobin-based oxygen carrier

**DOI:** 10.1038/s41598-020-68190-0

**Published:** 2020-07-09

**Authors:** Donald A. Belcher, Alfredo Lucas, Pedro Cabrales, Andre F. Palmer

**Affiliations:** 1 William G. Lowrie Department of Chemical and Biomolecular Engineering, The Ohio State University, Columbus, OH 43210 USA; 2 Department of Bioengineering, University of California San Diego, La Jolla, CA 92093 USA

**Keywords:** Cancer imaging, Cancer microenvironment, Cancer therapy, Skin cancer

## Abstract

Administration of hemoglobin-based oxygen carriers (HBOCs) into the systemic circulation is a potential strategy to relieve solid tumor hypoxia in order to increase the effectiveness of chemotherapeutics. Previous computational analysis indicated that the oxygen (O_2_) status of the tumor and HBOC O_2_ affinity may play a role in increased O_2_ delivery to the tumor. However, no study has experimentally investigated how low- and high-affinity HBOCs would perform in normoxic and hypoxic tumors. In this study, we examined how the HBOC, polymerized human hemoglobin (PolyhHb), in the relaxed (R) or tense (T) quaternary state modulates O_2_ delivery to hypoxic (FME) and normoxic (LOX) human melanoma xenografts in a murine window chamber model. We examined microcirculatory fluid flow via video shearing optical microscopy, and O_2_ distributions via phosphorescence quenching microscopy. Additionally, we examined how weekly infusion of a 20% top-load dose of PolyhHb influences growth rate, vascularization, and regional blood flow in the FME and LOX tumor xenografts. Infusion of low-affinity T-state PolyhHb led to increased tissue oxygenation, decreased blood flow, decreased tumor growth, and decreased vascularization in hypoxic tumors. However, infusion of both T-state and R-state PolyhHbs led to worse outcomes in normoxic tumors. Of particular concern was the high-affinity R-state PolyhHb, which led to no improvement in hypoxic tumors and significantly worsened outcomes in normoxic tumors. Taken together, the results of this study indicate that the tumor O_2_ status is a primary determinant of the potency and outcomes of infused PolyhHb.

## Introduction

Unregulated angiogenesis and rapid cell proliferation in the tumor microenvironment result in decreased blood flow and oxygen (O_2_) delivery^1^. Under these conditions, cancer cells adapt to the hypoxic environment via activation of hypoxia-inducible factors, HIF-1 and HIF-2^2^. These adaptations to chronic hypoxia are associated with metabolic reprogramming, angiogenesis, epithelial-mesenchymal transition, metastasis, and resistance to radiation and chemotherapy^3,4^. Furthermore, many forms of cancer therapy require reactive oxygen species (ROS) to promote tumor suppression^5^. Thus, increasing O_2_ delivery to solid tumors is a promising target for cancer therapy.

Due to their ability to modulate O_2_ delivery from the circulatory system, hemoglobin-based oxygen carriers (HBOCs) are promising O_2_ therapeutics that may increase the effectiveness of chemotherapy^6–12^. However, HBOCs are still not clinically approved despite decades of research^13,14^. The elevated renal toxicity and hypertension associated with previous generations of commercial HBOCs have mainly been attributed to this delay in development. Current improvements in reactor design and product purification are now able to exclude the low molecular weight (MW) HBOC fractions (< 250 kDa ) that contributed to the toxicity of early generation HBOCs^15–18^.

The chemical modifications that are necessary to make HBOCs safe for infusion typically alter the HBOC O_2_ affinity^16,17,19–21^. For example, conjugating polyethylene glycol to the surface of hemoglobin (Hb) results in an increased O_2_ affinity (P_50_: 5–6 mm Hg) compared to unmodified Hb^22,23^. Alternatively, commercial polymerized Hbs (PolyHbs) prepared via glutaraldehyde or O-raffinose crosslinking in the tense (T) quaternary state all have significantly lower O_2_ affinity (P_50_: 30–40 mm Hg) compared to unmodified Hb^24–26^. Recently, we demonstrated that the O_2_ affinity of PolyHbs could be controlled by polymerizing the Hb under fully oxygenated or deoxygenated conditions^16,17,21^. By fully oxygenating the Hb during polymerization, the PolyHb is effectively locked into the high O_2_ affinity, relaxed quaternary state (R-state). Alternatively, polymerizing the Hb while deoxygenated locks the PolyHb into the low O_2_ affinity, T-state.

Previously, we computationally determined that alterations in the O_2_ affinity of the HBOC impact how much O_2_ an HBOC will supply to the surrounding tumor tissue^20,27^. From the results of these studies, we anticipate that R-state PolyHb and T-state PolyHb should increase O_2_ delivery to hypoxic tissue. Whereas in normoxic tissue, T-state PolyHb should increase oxygenation. To date, no study has examined how infusing an HBOC may impact the growth and vascularization of hypoxic and normoxic tumors. Previous studies only examined hypoxic tumors or a single class of HBOC^28–33^. Understanding how both normoxic and hypoxic tumors respond to different modes of enhanced O_2_ delivery is vital to defining how these materials would be applied clinically.

In this work, we prepared high MW polymerized human Hbs (PolyhHbs) in either the T- or R-state. We then observed how each of these materials modulate O_2_ transport within hypoxic (FME) and normoxic (LOX) human melanoma xenografts. By using the LOX melanoma cell line, we are able to generate normoxic tumors to compare with the hypoxic tumors^34,35^. By using phosphorescence quenching microscopy (PQM) on the microcirculatory environment observed within window chamber models, we explored how PolyhHbs modulate blood flow and O_2_ transport in vivo. Additionally, we examined how weekly infusions of PolyhHb impact tumor growth and vascularization. With these results, we are better able to define how the oxygenation status of the tumor and the O_2_ affinity of an HBOC will impact modulation of O_2_ delivery.

## Results

### Biophysical properties of PolyhHbs

**Figure 1:**
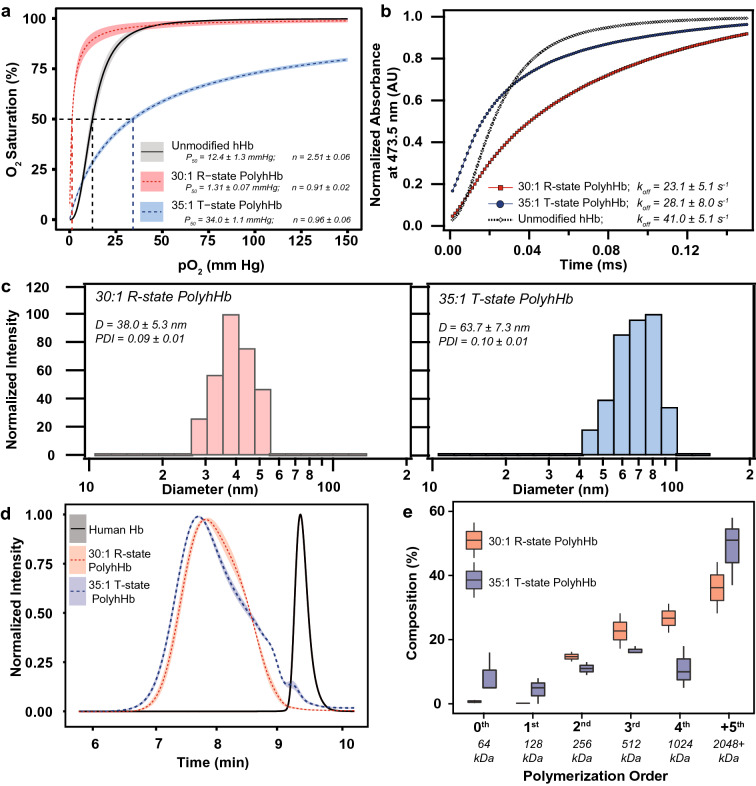
Biophysical properties of unmodified hHb, 35:1 T-state PolyhHb, and 30:1 R-State PolyhHb. (**A**) OECs for PolyhHb and hHb. (**B**) Comparison of the kinetic time course for deoxygenation in the presence of 1.5 mg/mL sodium dithionite for hHb, 30:1 R-state PolyhHb, and T-state PolyhHb. For OECs (**A**), The shaded region represents the 95% confidence interval for each quaternary state with three runs per sample. For deoxygenation (**B**), the experimental data shows an average of 10–15 kinetic traces. For deoxygenation, the reactions were monitored at 437.5 nm and 20 °C in 0.1 M pH 7.4 PBS. Symbols represent experimental data, and corresponding lines of the same color represent curve fits. (**C**) Representative intensity distributions of the hydrodynamic diameter of 30:1 R-state and 35:1 T-state PolyhHb. (**D**) Normalized SEC intensity distributions of R-state and T-state PolyhHb compared to unmodified hHb. The shaded region represents the 95% confidence interval for the average of the produced PolyhHb. (**E**) Polymer order distribution for 35:1 T-state and 30:1 R-state PolyhHb. Polymer distribution was calculated on a percent by heme basis via analysis of the 413 nm absorbance wavelength. The corresponding approximate sizes of the polymer orders are shown below each group.

The resulting properties of the PolyhHbs used in this study are shown in Fig. 1. The metHb levels of the resulting solutions were 5.8 ± 0.8% for 35:1 T-state PolyhHb and 4.2 ± 0.9% for 30:1 R-state PolyhHb. The concentration of both solutions was maintained at 100 ± 1.5 mg/mL. Polymerization under fully oxygenated conditions resulted in a significant increase in O_2_ affinity for 30:1 R-state PolyhHb (P_50_ = 1.31 ± 0.07 mmHg) compared to hHb (P_50_ = 12.4 ± 1.3 mm Hg). In contrast, polymerization under completely deoxygenated conditions significantly decreased O_2_ affinity (P_50_ = 34 ± 1.1 mmHg). After polymerization, the rate of O_2_ release is significantly slower when compared to unmodified hHb. The 30:1 R-state PolyhHb (D = 38 ± 5.3 nm) was smaller than 35:1 T-state PolyhHb (D = 63.7 ± 7.3 nm). Despite being larger on average, 35:1 T-state PolyhHb contained significantly more low MW species (0th, 1st order) compared to 30:1 T-state PolyhHb.

### Hematological and blood gas parameters

Changes in hematological and blood gas parameters are displayed in Table 1. There were no differences in systemic blood parameters at baseline compared to healthy mice (no implanted tumor). Infusion of 35:1 T-state and 30:1 R-state PolyhHb significantly reduced the hematocrit. There was no significant difference between either the hematocrit or PolyhHb in the plasma after infusion of either 35:1 T-state or 30:1 R-state PolyhHb. Infusion of both 35:1 T-state PolyhHb and 30:1 R-state PolyhHb resulted in significant increases in MAP. In mice implanted with FME tumors, infusion of 30:1 R-state PolyhHb led to a significant decrease in HR compared to the Baseline. Similarly, in mice implanted with FME tumors, there was a significant increase in PaO_2_ after infusion of 30:1 R-state PolyhHb compared to the baseline and 35:1 T-state PolyhHb treatment groups. In both tumors, infusion of 30:1 R-state PolyhHb led to a significant increase in PaCO_2_ and a significant decrease in pH compared to both the baseline and 35:1 T-state PolyhHb treatment groups.

**Table 1:** Hematological and blood gas parameters for healthy animals and animals implanted with FME or LOX tumor infused with a 20% top-load dose of either 35:1 T-state PolyhHb or 30:1 R-state PolyhHb.

Parameter	Healthy	FME human melanoma xenograft			LOX human melanoma xenograft		
		Baseline	35:1 T-state PolyhHb	30:1 R-state PolyhHb	Baseline	35:1 T-state PolyhHb	30:1 R-state PolyhHb
Hb (g/dL)	14.6 ± 0.2	14.6 ± 0.2^‡^	10.8 ± 0.4^†^	11.3 ± 0.5^†^	14.6 ± 0.2^‡^	10.9 ± 0.2^†^	10.9 ± 0.2^†^
Plasma PolyhHb (g/dL)	–	–	1.4 ± 0.1	1.3 ± 0.1	–	1.3 ± 0.1	1.5 ± 0.1
MAP (mmHg)	105 ± 7	104 ± 6^‡^	122 ± 4^†^	126 ± 9^†^	105 ± 8^‡^	128 ± 5^†^	125 ± 9^†^
HR (beats/min)	517 ± 28	514 ± 26	492 ± 25	469 ± 28^†^	518 ± 25	484 ± 27	496 ± 22
PaO_2_ (mmHg)	106 ± 5	105 ± 5	97 ± 7	113 ± 8^†‡^	106 ± 7	100 ± .3	107 ± 7
PaCO_2_ (mmHg)	34.7 ± 1.9	35.0 ± 2.2	35.1 ± 2.0	32.0 ± 1.3^†‡^	34.5 ± 1.6	34.9 ± 1.5	32.3 ± 2.5^†‡^
pH	7.29 ± 0.09	7.32 ± 0.10^‡^	7.38 ± 0.09^†^	7.25 ± 0.12^†‡^	7.29 ± 0.07^‡^	7.35 ± 0.10^†^	7.26 ± 0.10^†‡^

^†^*P* < 0.05 compared to baseline. ^‡^*P* < 0.05 compared to 35:1 T-state PolyhHb (n = 6 mice).

### Tissue O_2_ tension

**Figure 2:**
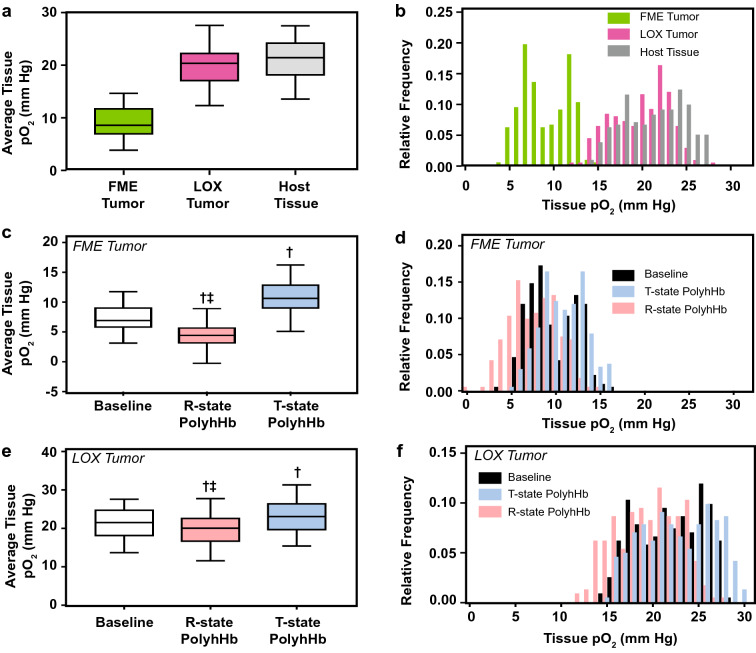
Average tissue pO_2_ and distribution of measured tissue pO_2_ in host tissue, and in hypoxic, and normoxic tumor xenografts as measured via phosphorescence quenching microscopy in the chamber window model. This figure shows the (**A**, **C**) average pO_2_s and (**B**, **D**) frequency of tissue pO_2_ for (**A**, **B**) hypoxic FME human melanoma and (**C**, **D**) normoxic LOX human melanoma tumor xenografts. Data is shown for the baseline and after infusion of 30:1 R-state PolyhHb and 35:1 T-state PolyhHb. ^†^*P* < 0.05 compared to baseline. ^‡^*P* < 0.05 compared to 35:1 T-state PolyhHb. (n = 5 mice).

Changes in tissue O_2_ tension in both hypoxic (FME) and normoxic (LOX) tumors are depicted in Fig. 2. The average tissue pO_2_ of hypoxic (FME) tumors was significantly lower than the average tissue pO_2_ in normoxic (LOX) tumors and host tissue. Infusion of 30:1 R-state PolyhHb significantly decreased tissue oxygenation in both normoxic (LOX) and hypoxic (FME) tumors compared to baseline and following infusion of 35:1 T-state PolyhHb. In contrast, infusion 35:1 T-state PolyhHb led to significant increases in average tissue pO_2_ in both tumors.

### Microhemodynamics

**Figure 3:**
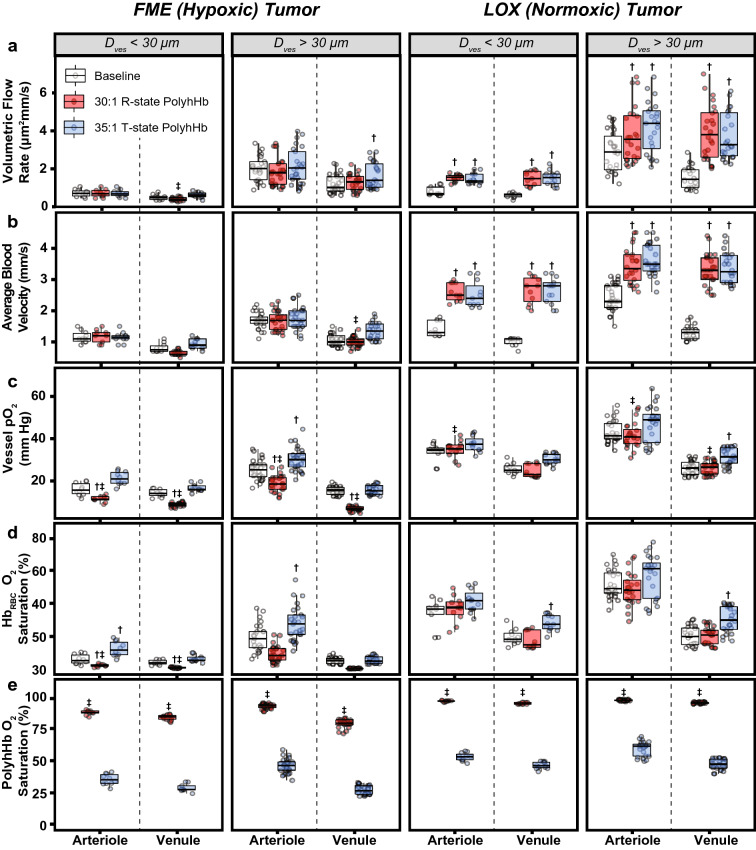
Results from the analysis of how PolyhHb modulates fluid and O_2_ transport as measured in a murine chamber window model containing either hypoxic or normoxic tumors. This figure shows (**A**) volumetric flow rate through a blood vessel, (**B**) average blood velocity through the blood vessel, (**C**) average pO_2_ in the blood vessel, (**D**) O_2_ saturation of Hb in RBCs, and (**E**) O_2_ saturation of PolyhHb in the plasma in hypoxic (FME) and normoxic (LOX) tumors. Vessels have been grouped by whether they flow into (arteriole) or out of (venule) the tumor along with whether the vessel is small (< 30 μm) or large (> 30 μm). ^†^*P* < 0.05 compared to baseline. ^‡^P < 0.05 compared to 35:1 T-state PolyhHb. (n = 5 mice).

Changes in the arteriolar and venular blood flow in both hypoxic (FME) and normoxic (LOX) tumors are depicted in Fig. 3A, B. Vessel diameters were separated into two groups for this analysis: one group with diameters less than 30 μm and another group with diameters greater than 30 μm. In hypoxic tumors, infusion of 30:1 R-state PolyhHb led to a significant reduction in the volumetric flow rate in small diameter (${D}_{ves}$ < 30 μm) venules. Infusion of 35:1 T-state PolyhHb led to significant increases in the flow rate in large diameter (${D}_{ves}$ > 30 μm) venules. In normoxic tumors, infusion of both PolyhHb species led to significant increases in tumor perfusion in all blood vessels. In general, blood flow velocity in normoxic tumors was significantly higher than blood flow in hypoxic tumors.

### Intravascular pO_2_ and O_2_ saturations

Changes in intravascular pO_2_, O_2_ saturation of Hb in RBCs, and O_2_ saturation of PolyhHbs are shown in Fig. 3C–E. The O_2_ saturation of Hb in RBCs was always significantly greater in the normoxic tumor compared to the hypoxic tumor. Infusion of 30:1 R-state PolyhHb led to significant reduction in blood O_2_ saturation in hypoxic tumors compared to both the control and infusion of 35:1 T-state PolyhHb. This is coupled with a corresponding significant (*P* < 0.05) decrease in O_2_ saturation of Hb in RBCs compared to the control and infusion of 35:1 T-state PolyhHb. Infusion of 35:1 T-state PolyhHb leads to a significant increase in intravascular pO_2_ in large diameter (${D}_{ves}$ > 30 μm) arterioles in the hypoxic tumor and large diameter (${D}_{ves}$ > 30 μm) venules in the normoxic tumor. Additionally, infusion of 35:1 T-state PolyhHb significantly (*P* < 0.05) increased O_2_ saturation of Hb in RBCs in the arterioles of hypoxic tumors and the venules of normoxic tumors. As anticipated, the O_2_ saturation of 30:1 R-state PolyhHb was always significantly greater than the O_2_ saturation of 35:1 T-state PolyhHb.

### Oxygen extraction fraction (OEF)

**Figure 4:**
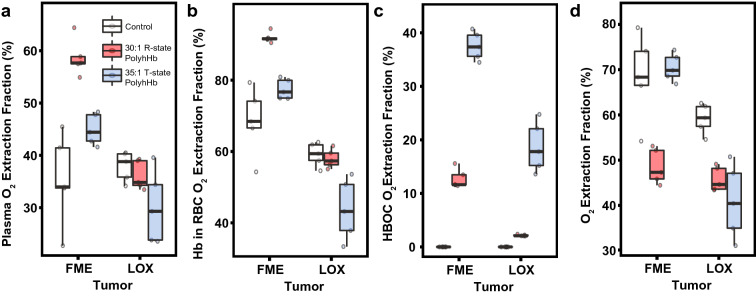
Oxygen extraction fraction from various species as measured in a murine chamber window model containing either hypoxic or normoxic tumors. This figure displays the oxygen extraction fraction (OEF) as a percentage of total inlet oxygen (O_2_) for (**A**) O_2_ dissolved in the plasma, (**B**) O_2_ bound to Hb in RBCs, (**C**) O_2_ bound to PolyhHbs, and (**D**) total O_2_ from all O_2_ carrying species in hypoxic (FME) and normoxic (LOX) tumors. ^†^*P* < 0.05 compared to baseline. ^‡^*P* < 0.05 compared to treatment with 35:1 T-state PolyhHb. (n = 5 mice).

The changes in the calculated plasma OEF, Hb in RBC OEF, PolyhHb OEF, and overall OEF in hypoxic (FME) and normoxic (LOX) tumors are displayed in Fig. 4. Infusion of 30:1 R-state PolyhHb significantly increased the amount of O_2_ extracted from plasma and RBCs in hypoxic (FME) tumors. Infusion of 35:1 T-state PolyhHb led to a significant reduction in the OEF from plasma and Hb in RBCs. The OEF from 35:1 T-state PolyhHb was significantly greater than the O_2_ extraction from 30:1 R-state PolyhHb. Infusion of 30:1 R-state PolyhHb decreased overall OEF in both hypoxic (FME) and normoxic (LOX) tumors. Infusion of 35:1 T-state PolyhHb led to significant reduction in overall OEF in LOX tumors; however, it did not lead to any significant changes in the OEF in FME tumors.

### Tumor growth

**Figure 5:**
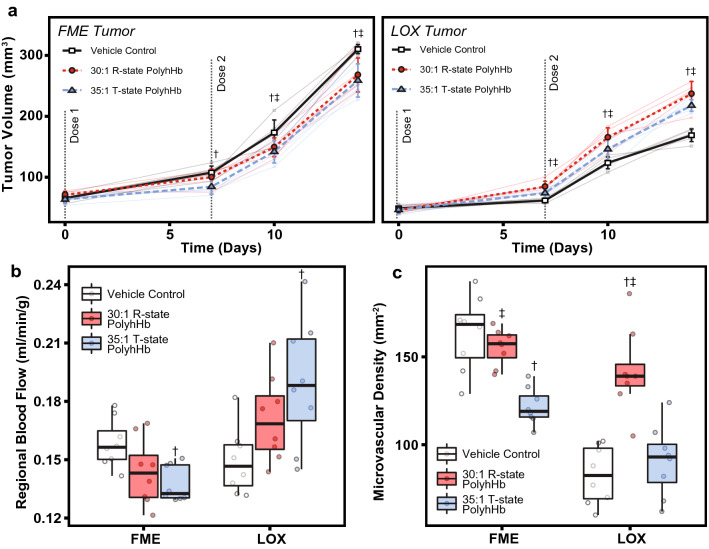
Tumor properties after weekly PolyhHb infusions. (**A**) Volume expansion of hypoxic and normoxic tumor xenografts over two weeks after weekly infusion of 35:1 T-state, 30:1 R-state PolyhHb, or the vehicle control to (LEFT) hypoxic FME and (RIGHT) normoxic LOX human melanoma tumor xenografts. Grey lines connect tumor growth measured in the same animal. (**B**) Microvascular density measured at the end of tumor growth after weekly administration of PolyhHb. (**C**) Regional blood flow after weekly administration of PolyhHb. (**D**) Microvascular density (represented as the density of competent vessels in the viewing area) after weekly infusion of PolyhHb or the vehicle control. ^†^*P* < 0.05 compared to baseline. ^‡^*P* < 0.05 compared to treatment with 35:1 T-state PolyhHb.

In addition to examining how PolyhHb altered microvascular O_2_ transport in tumors, we also examined how a low volume weekly dose of 30:1 R-state PolyhHb and 35:1 T-state PolyhHb would impact tumors implanted into tissue. The effects of weekly 20% top-load doses of 30:1 R-state PolyhHb and 35:1 T-state PolyhHb on the volumes of FME and LOX tumor xenografts are shown in Fig. 5. The approximate rate of radial expansion was approximately 5.5 μm/h for FME (hypoxic) tumors and approximately 4.1 μm/h for LOX (normoxic) tumors. After top-load infusion of both T-state and R-state PolyhHb, there is a significant reduction in the growth of the hypoxic FME tumor. Infusion of 35:1 T-state PolyhHb resulted in a significant reduction in FME tumor growth (20%) compared to 30:1 R-state PolyhHb (9.6%).

### Tumor vasculature

We were also interested in experimentally observing how periodic infusions of PolyhHb solutions would alter properties associated with microvascular mass transport. Regional blood flow (RBF) was analyzed with fluorescent microsphere perfusion, and microvascular density (MVD) was estimated via tissue histology. The results of these studies are shown in Fig. 5B, C. In hypoxic FME tumor xenografts, infusion of 35:1 T-state PolyhHb led to a significant decrease in both RBF and MVD. In normoxic LOX tumor xenografts, infusion of T-state PolyhHb led to a significant increase in RBF to the tumor compared to baseline.

## Discussion

The principal finding of this study is that the O_2_ status of tumors has a strong effect on the effects of PolyhHb coadministration. These results may help explain some of the previous negative results that occurred in rhabdomyosarcomas^28^.

In this study, we investigated how infusion of PolyhHb influences microcirculatory fluid and O_2_ transport within a murine chamber window model. In general, infusion of 35:1 T-state PolyhHb led to increased tumor perfusion and O_2_ delivery. In contrast, infusion of 30:1 R-state PolyhHb decreased overall fluid and O_2_ transport. This can be attributed to two factors: (1) 30:1 R-state PolyhHb had extremely high O_2_ affinity, and (2) 30:1 R-state PolyhHb was slightly hypertensive. After infusion of 30:1 R-state PolyhHb there was a significant increase in MAP and PaO_2_ coupled with a decrease in PaCO_2_, HR, and pH. In comparison, infusion of 35:1 T-state PolyhHb primarily increased MAP. These increases in MAP is consistent with previous HBOCs containing low MW species^36,37^.

In normoxic tumors, the hemodulution effect of PolyhHb infusion led to significant increases in tumor perfusion. Despite this increase in blood perfusion after the infusion of R-state PolyhHb, the average tissue pO_2_ decreased. This decrease is likely a result of the relatively low amount of O_2_ extracted from R-state PolyhHb (2.2 ± 0.1% ), which failed to make up for the lack of O_2_ carrying capacity from the diluted blood. In comparison, 18.7 ± 4.7% of the total available O_2_ from T-state PolyhHb was extracted, which offset the O_2_ extraction from both Hb in RBCs and dissolved O_2_ in plasma.

In hypoxic tumors, the percentage of O_2_ extracted from 30:1 R-state PolyhHb only slightly increased (12.8 ± 1.8%) compared to the percentage of O_2_ extracted from 35:1 T-state PolyhHb (37.5 ± 2.6%). This slight increase in O_2_ extraction from R-state PolyhHb does not fully offset the O_2_ demand from O_2_ dissolved in plasma and Hb in RBCs. When the decrease in O_2_ supply from R-state PolyhHb is coupled with reduced blood flow resulting from vasoconstriction, overall O_2_ delivery is significantly reduced. This requirement is instead offset by significant increases in the O_2_ extracted by the dissolved O_2_ in the plasma and Hb in RBCs.

In this study, we found that the infusion of PolyhHb led to relatively minor changes in the growth rate of the hypoxic (FME) tumors. However, it appears that this low dosage was unable to replicate the significant reduction in tumor growth (40%) that was previously observed in a triple-negative breast cancer model^20^. This is likely because the dose volume and dose frequency were too low to result in an appreciable effect on tumor growth. For this study, the PolyhHb was delivered weekly; however, previous studies of the pharmacokinetics of similar PolyHbs indicate that these PolyhHbs have a half-life of only 24 h^15^. Taking this into account, the tumors were only exposed to the O_2_ modulating effect of PolyhHb for only 25% of the week. Increasing the dosing frequency to once every 2 to 3 days may increase the relative effect.

Unfortunately, infusion of both the T-state and R-state PolyhHb solutions led to a significant increase in tumor growth for normoxic LOX tumors. Infusion of 30:1 R-state PolyhHb led to a 40% increase in tumor volume after the 14-day treatment regime. This is likely because both 35:1 T-state PolyhHb and 30:1 R-State PolyhHb have higher O_2_ affinity compared to mouse Hb in RBCs (P_50_ = 42 mm Hg). Because of this relative increase in O_2_ affinity, we anticipate that less O_2_ may be delivered under normoxic conditions, which could decrease host cell survival in the tumor periphery. We anticipate that when applied to a model that more accurately represents human physiology, T-state PolyhHb might decrease the tumor growth rate due to its lower O_2_ affinity compared to human Hb in RBCs (P_50_ = 26 mm Hg). However, we may also observe further increases in tumor growth due to the increased supply of O_2_ to normoxic tumors.

Despite observing a growth delay after infusion of 30:1 R-state PolyhHb, infusion of R-state PolyhHb did not lead to significant decreases in RBF or MVD. This is likely because the low dose frequency and high O_2_ affinity of R-state PolyhHb were insufficient to trigger an anti-angiogenic response in FME tumors. Baseline values for RBF in hypoxic FME tumors [0.16 ± 0.02 mL/(min g)] and normoxic LOX tumors [0.15 ± 0.02 mL/(min g)] are at the upper range of the values measured for other tumors experimentally^38–40^. In the hypoxic (FME) tumors, we observed decreases in MVD after delivery of 35:1 T-state PolyhHb. This decrease in vessel formation indicates an increase in O_2_ delivery. In contrast, we observed a significant increase in MVD after weekly infusions of 30:1 R-state PolyhHb in normoxic (LOX) tumors. This is consistent with a decrease in O_2_ delivery, which may lead to more aggressive tumor growth and increased angiogenesis. In fact, the MVD of the normoxic LOX tumor after infusion of 30:1 R-state PolyhHb is remarkably similar to the measured MVD within the baseline hypoxic (FME) tumor. This further supports the notion that within the normoxic tumor, R-state PolyhHb is not adequately delivering O_2_, which is in agreement with microvascular simulations performed previously^20^. Despite this, reduction in tumor growth has been previously observed after infusion of high O_2_ affinity HBOCs^41–43^. Therefore, this decrease in tumor growth may be due to other factors in the environment including production of reactive O_2_ species (ROS)^44^ and nitric oxide (NO) scavenging^45^. This faster rate of metHb formation will lead to the increased production of ROS which can induce oxidative injury to the tumor mass. This is especially important to consider given that HBOCs can scavenge NO^46^ and can oxidize and produce ROS^47^ species in vivo. Future studies should investigate these mechanisms in more detail by directly observing changes in hypoxia inducible factors and downstream proteins when working with high O_2_ affinity HBOCs.

## Conclusions

The results from this study indicate that low-dose, infrequent infusions of R-state PolyhHb is not suitable for oxygenating both hypoxic and normoxic melanomas. In general, treatment of normoxic tumors with either high- or low O_2_ affinity PolyhHbs aggravated tumor growth and angiogenesis. In contrast, T-state PolyhHbs significantly increased O_2_ supply to hypoxic tumors. These results encourage the use of low O_2_ affinity PolyhHbs with reduced cooperativity to hypoxic tumors. Additionally, this further emphasizes the need to fully characterize how different tumor types respond to modulating O_2_ delivery with HBOCs.

## Methods

### Polymerized hemoglobin synthesis and analysis

Human Hb (hHb) used in these studies was first purified from human red blood cells (RBCs) as described previously^48^. PolyhHb was produced using methods described previously^17^. In brief, the resulting hHb solution was polymerized with glutaraldehyde while fully oxygenated or deoxygenated to form either R-state or T-state PolyhHb, respectively. The resulting PolyhHbs were first clarified on a 0.2 μm hollow fiber filter. After clarification, the PolyhHb solutions were diafiltered on a 100 kDa hollow fiber filter into a modified Ringer’s lactate buffer to remove the low MW PolyhHb/hHb species. The cyanomethemoglobin method was used to measure the Hb concentration and the metHb level of hHb/PolyhHb solutions^49,50^. The size distribution of PolyhHb, by particle volume, was measured using dynamic light scattering (DLS) (Brookhaven Instrument Inc. BS-200M, Holtsville, NY). The O_2_-hHb/PolyhHb equilibrium binding curves were measured using a Hemox Analyzer (TCS Scientific Corp., New Hope, PA). The hHb/PolyhHb kinetics of O_2_ offloading (${k}_{off,{O}_{2}}$) were measured with an Applied Photophysics SF-17 microvolume stopped-flow spectrophotometer (Applied Photophysics Ltd., Surrey, United Kingdom) using protocols previously described by Rameez and Palmer^51–53^. The MW distribution was estimated using an Acclaim SEC-1000 column (Thermo Scientific, Waltham, MA) on a Thermo Scientific Dionex Ultimate UHPLC system using previously described methods^17,54^.

### Dorsal chamber window model

Adult female 8- to 10-week old female BALB/c-nu/nu mice were used for the xenografted tumors according to protocols approved by the University of California San Diego Animal Care and Use Committee. Mice were instrumented with dorsal chamber windows as described previously^55,56^. This experimental model is an excellent system to observe O_2_ delivery as it is highly sensitive to changes in O_2_ supply^57^. Additionally, this model is especially useful when examining changes in microvascular pO_2_ distributions in the unanesthetized state. Human melanomas (FME and LOX) were initiated by implanting a 200–500 μm xenograft into the fascial side of the intact skin layer of the chamber window model. FME and LOX cells were generously donated by Micro-Target Dynamic Therapy (San Diego, CA). The FME and LOX human melanoma cell lines were originally developed by the Rofstad Group at the Institute for Cancer Research (Oslo University Hospital, Norway)^34,35^. After implantation of the xenografts, tumors were allowed to grow for 7 days before analysis. Mice were divided into two groups based on the implanted human tumor cell lines (FME or LOX). Each of these groups was further subdivided into three cohorts: (1) an unsupplemented baseline, (2) infusion of 30:1 R-state PolyhHb, and (3) infusion of 35:1 T-state PolyhHb. At this point, mice underwent a 20% top load (20% of the mouse blood volume calculated by weight) infusion of a 30:1 R-state PolyhHb or a 35:1 T-state PolyhHb at a concentration of 100 mg/mL. PolyhHb was infused via tail vein injection. After infusion, the animal was placed into a restraining tube. Once in the tube, the protruding chamber window was fixed to a microscopic stage of a BX51WI intravital microscope (Olympus, New Hyde Park, NY). Tissue images were then projected to a 4,815 charge-coupled device camera (Cohu Industries, Poway, CA). A LUMPFL-WIR × 40 numerical 0.8 aperture water immersion objective (Olympus, New Hyde Park, NY) was used to carry out the measurements. Mean arterial pressure (MAP) and heart rate (HR) were recorded using a pressure transducer connected to the femoral artery catheter using an MP-150 system (BIOPAC Systems, Goleta, CA). Arterial blood was collected in heparinized capillary tubes and immediately analyzed for PaO_2_, PaCO_2_, and pH using a 248 Blood Chemistry Analyzer (Bayer, Norwood, MA). Total Hb was measured spectrophotometrically using a B-hemoglobin Hemocue (Stockholm, Sweden). Plasma Hb was measured from plasma collected after the capillary tube was centrifuged.

### Phosphorescence quenching microscopy (PQM)

Phosphorescence quenching microscopy (PQM) was used to analyze the O_2_ distribution in the tissue and vascular space, as described previously^58^. This high-resolution method allows us to resolve the pO_2_ of arterioles and venules within the growing tumor. To determine the pO_2_ in this method, we measure the decay rate of the excited palladium-mesotetra-(4-carboxyphenyl)porphyrin (Frontier Scientific Porphyrin Products, Logan, UT) bound to albumin. We then used the measured fluorescence lifetime (${\tau }_{p}$), fluorescence lifetime in the absence of O_2_ (${\tau }_{p,0}$), and fluorescence quenching rate constant (${k}_{q}$) to calculate the pO_2_ using the Stern–Volmer equation, as shown in Eq. (1)^59^.

1 $$ pO_{2} = \frac{{\left( {\frac{{\tau_{p,0} }}{{\tau_{p} - 1}}} \right)}}{{\tau_{p,0} k_{q} }} $$

The probe was injected intravenously 10 min before pO_2_ distributions were measured to allow time for the phosphorescent probe to circulate and diffuse into the chamber window model. The exposed tissue within the chamber window was then excited with 420 nm wavelength light. To acquire ${\tau }_{p}$, the 680 nm emitted phosphorescence signal was collected. Because this method is relatively independent of the probe concentration, we were also able to measure extravascular tissue pO_2_.

### Microvascular hemodynamics

To observe changes in the arteriole and venule diameter, we used a video image shearing method to determine blood vessel diameter^60^. Center-line velocities of arterioles and venules were measured with a 102B Vista Electronics photo-diode velocity tracker (San Diego, CA) using a cross-correlation method. Volumetric flow rate ($Q$) through the arterioles and venules was then calculated using the radius of the vessel (${r}_{ves}$) and average fluid velocity $({\stackrel{-}{v}}_{f})$, as described in Eq. (2). For these calculations, we assume that fluid velocity profile was parabolic in arterioles and venules.

2 $$ Q = \pi \overline{v}_{f} r_{ves}^{2} $$

We are also able to calculate the oxygen extraction fraction (OEF) from the various species (dissolved oxygen, RBCs, PolyhHb) in solution by calculating the average mass flux of O_2_ into the tumor subtracted by the blood flow normalized average mass flux of O_2_ out of the tumor. The resulting O_2_ mass deficit is then divided by the average mass flux of O_2_ into the tumor as shown in Eq. (3).

3 $$ OEF = { }\frac{{\frac{{\sum \left[ {O_{2,in} } \right]_{i} Q_{in} }}{n} - \frac{{\sum \left[ {O_{2,out} } \right]_{i} Q_{out} { }}}{n} \times \frac{{\sum Q_{in} }}{{\sum Q_{out} }}}}{{\sum \left[ {O_{2,in} } \right]_{i} Q_{in} { }}} $$

### Tumor growth model

Similar to the chamber window model study, adult 8- to 10-week old female BALB/c-nu/nu mice were used for the xenografted tumors according to protocols approved by the University of California San Diego Animal Care and Use Committee. Approximately 4 × 10^5^ cells of the human melanoma cell lines FME and LOX were injected into the mouse flank. Mice were divided upon the tumor cell lines (FME or LOX). Each of these groups was further subdivided into three cohorts: (1) an unsupplemented baseline, (2) infusion of 30:1 R-state PolyhHb. and (3) infusion 35:1 T-state PolyhHb. Mice were infused with the 100 mg/mL PolyhHb solutions via tail vein injection of 20% of the mouse's blood volume once each week during the study. During tumor growth, the length of the tumor (${L}_{tumor}$) and width of the tumor (${W}_{tumor}$) were both measured to estimate tumor volume as shown in Eq. (4).

4 $$ V_{tumor} = \frac{{\pi L_{tumor} W_{tumor}^{2} }}{6} $$

### Tumor blood flow

Fluorescently labeled microspheres were used to estimate tumor blood flow in tumors as described previously^61^. In short, 15 μm diameter fluorescent microspheres (Molecular Probes, Eugene, OR) were suspended in saline. 100 μL of this solution was rapidly injected into the animal via the tail vein. Arterial reference samples were simultaneously withdrawn at a constant rate of 100 μL/min for 1 min through an inserted femoral catheter. At the end of the protocol, the mice were euthanized with a lethal dose of sodium pentobarbital. Tumor tissue was then digested in 1 M KOH solution for 24 h. Fluorescent dye was extracted with Cellosolve (Fisher Scientific, Pittsburgh, PA). The fluorescent signal was then measured using an LS 50B luminescence spectrometer (PerkinElmer Corp., Norwalk, CT). Regional blood flow proportional to the fraction of cardiac output was calculated by measuring the number of fluorescent microspheres in the tumor tissue relative to the total in the arterial reference samples.

### Tumor histopathology

Harvested tumors were fixed in PBS 4% paraformaldehyde, embedded in paraffin cases, cut into sections, and stained. Vascular density was assessed by counting the number of capillary profiles within a 0.8 mm^2^ field of view. Positive capillaries were only counted if a lumen and a brown staining endothelial cell were identified.

### Statistical analysis

All results are presented as the mean ± standard deviation. Statistically significant changes were analyzed with two-way ANOVA followed by post-hoc analysis using Tukey’s multiple comparison test when appropriate. All statistics were calculated with R (v 3.6.2). Results were considered statistically significant if *P* < 0.05.

### Ethics approval

All experimental protocols used to handle the mice were approved by the University of California San Diego Institutional Animal Care and Use Committee. The Hb used to prepare these materials was purified from expired RBCs donated from the Wexner Medical Center (Columbus, OH).

## Data Availability

The datasets generated during and/or analysed during the current study are available from the corresponding author on reasonable request.
